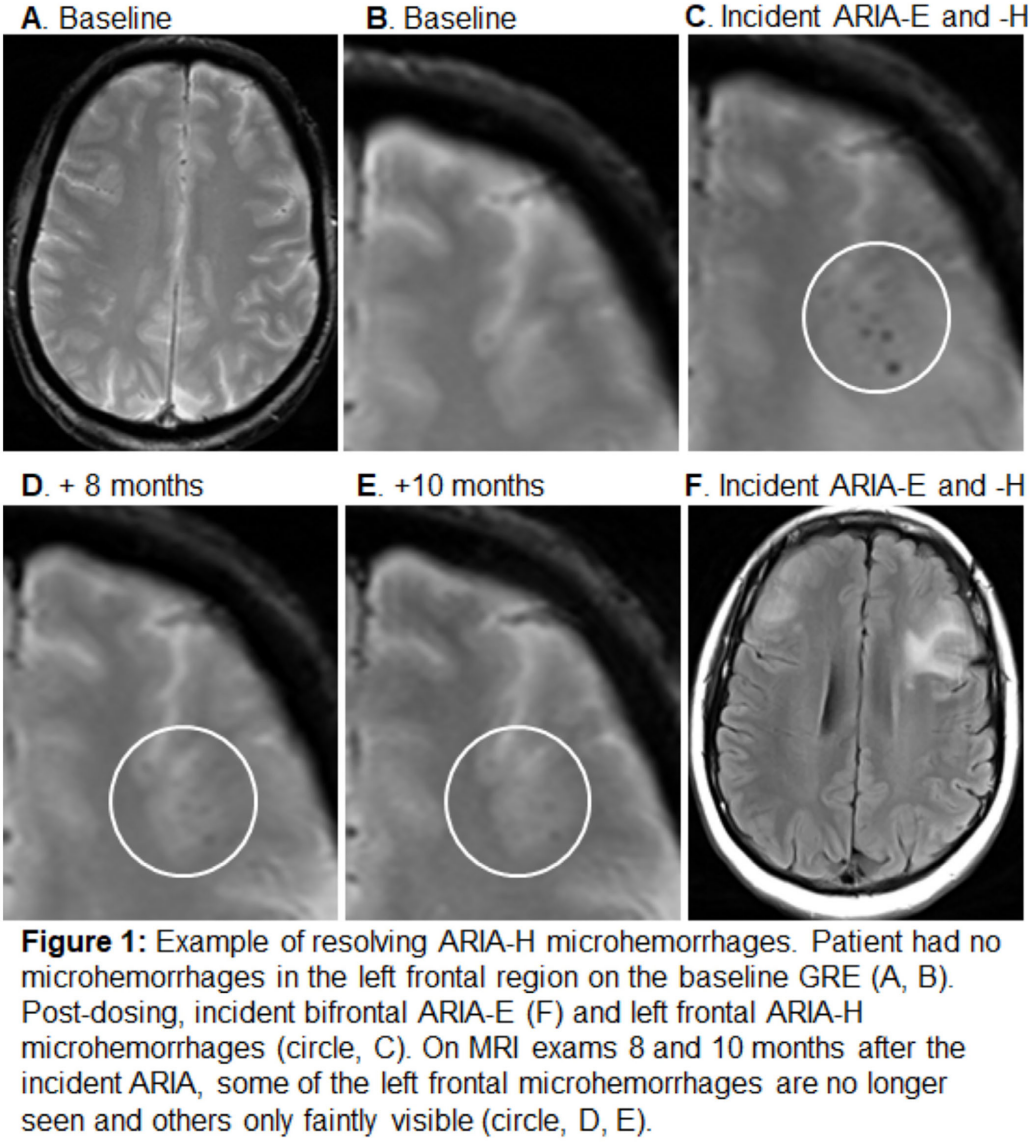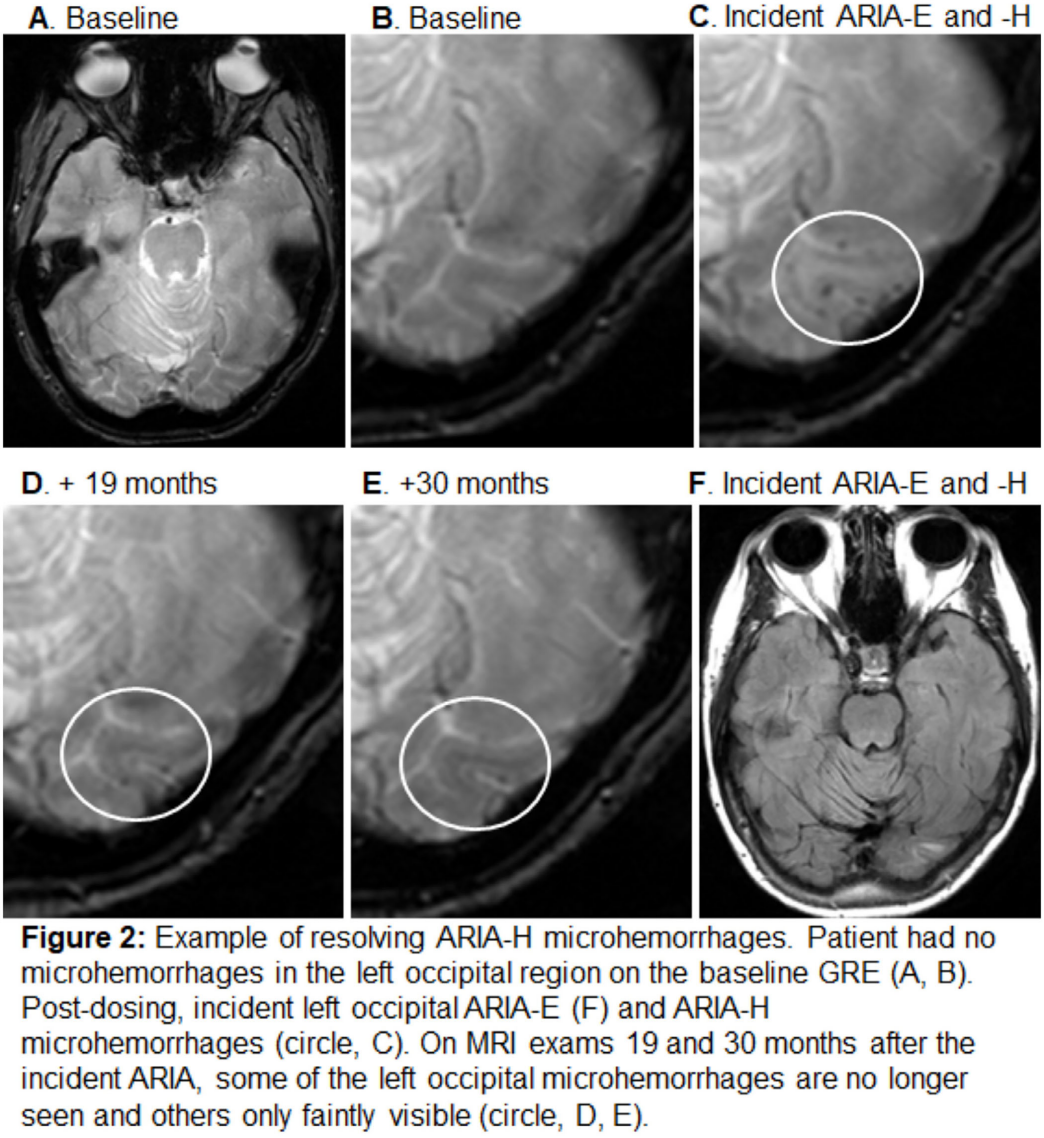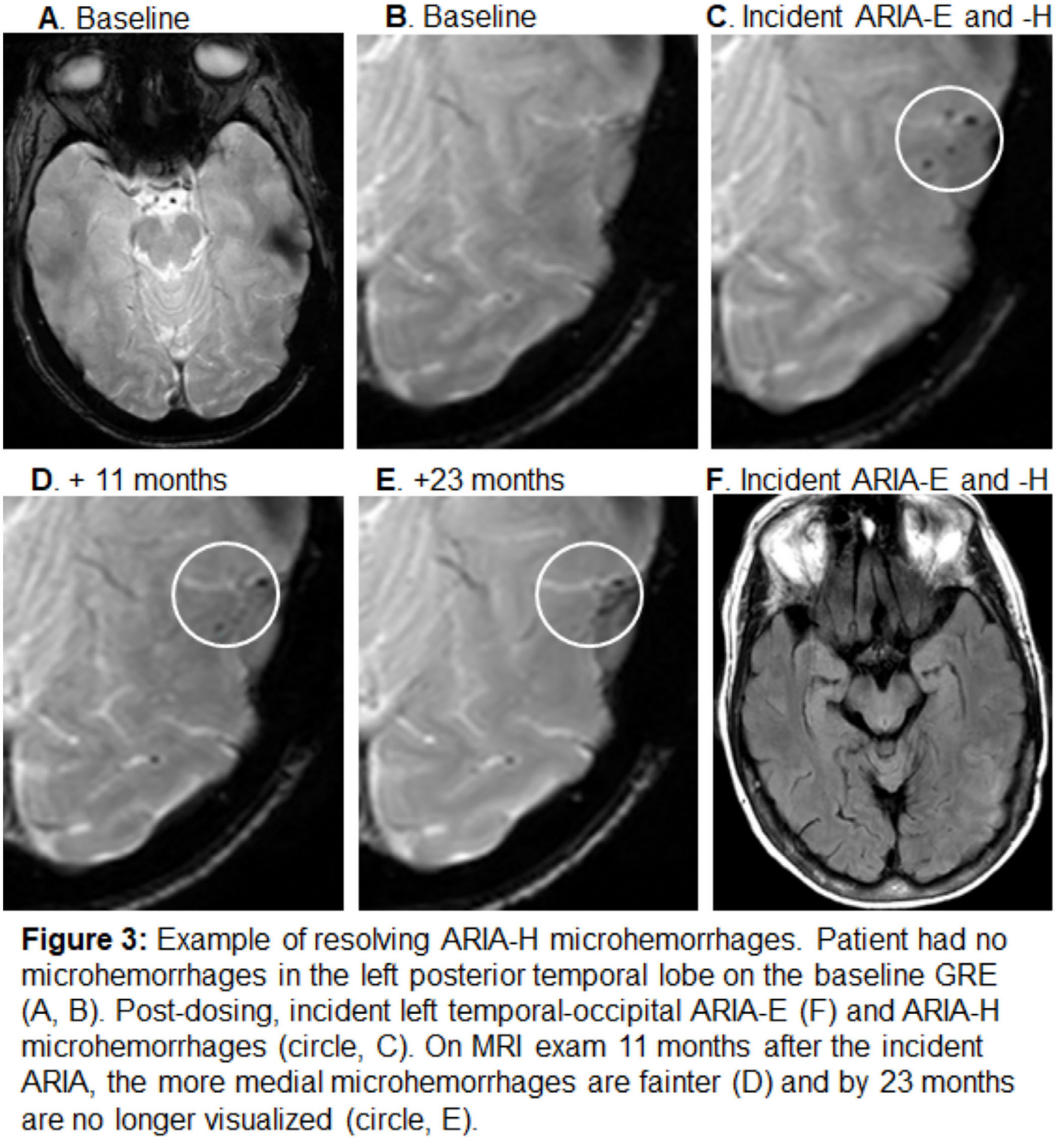# Disappearing ARIA‐H Microhemorrhages

**DOI:** 10.1002/alz70856_103166

**Published:** 2025-12-26

**Authors:** Petrice M Cogswell, Gregory M. Preboske, Gregory Klein, Tammie L.S. Benzinger, Randall J. Bateman, Clifford R. Jack, Dominantly Inherited Alzheimer's Network

**Affiliations:** ^1^ Mayo Clinic, Rochester, MN, USA; ^2^ Mayo Clinic, Radiology, Rochester, MN, USA; ^3^ Roche Pharma Research and Early Development, FHoffmann‐La RocheLtd, Basel, Switzerland; ^4^ Washington University in St. Louis, St. Louis, MO, USA; ^5^ Washington University School of Medicine, St. Louis, MO, USA

## Abstract

**Background:**

Amyloid‐Related Imaging Abnormalities (ARIA), an adverse event associated with Alzheimer's disease anti‐amyloid immunotherapies, occur in the form of edema or sulcal effusion (ARIA‐E) and microhemorrhages and superficial siderosis (ARIA‐H). Conventional teaching is that ARIA‐H is permanent hemosiderin staining of the brain. In clinical trials we have noticed that some ARIA‐H microhemorrhages that occur with regional ARIA‐E appear to later resolve. The goal of this study is to illustrate that, opposed to current understanding, some ARIA‐H microhemorrhages may resolve.

**Methods:**

We identified participants from the Dominantly Inherited Alzheimer's Network Trial Unit (DIAN‐TU) and open label extension (OLE) gantenerumab studies that had ARIA‐E and at least one ARIA‐H microhemorrhage. The T2* GRE sequence from all available MRI exams in a patient were co‐registered and reviewed by a board‐certified neuroradiologist. Individual microhemorrhages were tracked over the serial exams. We defined disappearing/resolving microhemorrhages as those appeared with or following ARIA‐E, were visualized on at least 2 MRI exams, and were subsequently not visualized on at least 2 MRI exams.

**Results:**

A total of 12 patients met inclusion criteria of which 5 (41%) had microhemorrhages that resolved over 10‐40 months follow‐up after the incident ARIA. These microhemorrhages occurred in clusters in the region of ARIA‐E, and only some of the regional microhemorrhages resolved over follow‐up. Examples are shown in Figures 1‐3.

**Conclusions:**

The disappearance (resolution) of ARIA‐H microhemorrhages is relevant to the management and continued dosing of patients receiving anti‐amyloid immunotherapies as this is guided by the presence of ARIA (including cumulative treatment emergent microhemorrhages) as well as clinical symptoms. A microhemorrhage number alone may not be indicative of the potentially dynamic nature of ARIA‐H.